# Effects of aquarobics on back pain, sleep, and memory in older women with chronic pain

**DOI:** 10.1097/MD.0000000000043199

**Published:** 2025-09-19

**Authors:** MoonSook Lee, Jiyoun Kim

**Affiliations:** a Department of Sports Science, Chungnam National University, Daejeon, South Korea; b Department of Exercise Rehabilitation, Gachon University, Incheon, South Korea.

**Keywords:** aquarobics, Back Pain Disability Index, chronic low back pain, older adult women, sleep disturbance, subjective memory impairment

## Abstract

**Background:**

Pain is a significant risk factor for cognitive decline in older adults. Chronic low back pain (CLBP) is particularly associated with memory impairment and decreased quality of life. This study aimed to examine the relationship between CLBP and memory impairment and to evaluate the effectiveness of aquarobic exercise as a non-pharmacological intervention.

**Methods:**

A total of 80 older women aged 65 years and above, with CLBP lasting >1 year, were recruited and randomly assigned to an experimental group (n = 68) or a control group (n = 84), using a 40:60 allocation to account for potential dropouts. The experimental group participated in 60-minute aquarobic sessions twice a week for 12 weeks, while the control group maintained a sedentary lifestyle. Participants’ characteristics included a mean age of 70.14 ± 1.48 years in the experimental group and 71.07 ± 1.04 years in the control group, with a mean CLBP duration of 8.68 ± 0.31 years and 8.71 ± 0.48 years, respectively. Data were analyzed using independent *t*-tests, repeated-measures analysis of variance, and structural equation modeling (SEM) to assess changes in mobility limitations, sleep disturbance, and subjective memory impairment.

**Results:**

After the intervention, the experimental group showed significant improvements across all measured variables. The Back Pain Disability Index significantly decreased (mean difference = –7.6, 95% CI: –10.5 to –4.9, *P* < .001), as did sleep disturbance (mean difference = –4.2, 95% CI: –6.8 to–1.5, *P* < .001) and subjective memory impairment (mean difference = –3.9, 95% CI: –6.1 to –1.7, *P* < .001). Structural equation modeling further revealed that the improvement in Back Pain Disability Index positively affected sleep and memory outcomes (*P* < .01), suggesting that physical pain relief mediates cognitive and psychological enhancement.

**Conclusion:**

Aquarobic exercise significantly improved functional mobility, sleep quality, and memory in older women with CLBP. These findings support aquarobics as an effective, holistic, and non-pharmacological intervention that enhances both physical and cognitive health in the elderly population.

## 1. Introduction

Cognitive decline is a growing public health concern worldwide, particularly in aging societies. With the global increase in life expectancy, the burden of age-related cognitive impairment, including mild cognitive impairment and dementia, has escalated significantly, prompting the urgent need for effective and accessible preventive strategies.^[1,2]^ Recent studies have reinforced that chronic musculoskeletal pain, especially in older adults, plays a significant role in the progression of cognitive dysfunction.^[3,4]^

Among musculoskeletal conditions, chronic low back pain (CLBP; ICD-10 code M54.50) is one of the most common conditions associated with movement limitations, disproportionately affecting older women due to sex-related physiological differences such as higher osteoporosis risk, lower muscle mass, and occupational exposure to domestic labor.^[5]^ According to recent reports from the World Health Organization (WHO), CLBP is significantly more prevalent in women than men, and its impact is most pronounced in older age groups. In particular, WHO (2019) noted that women experience a higher burden of disability from low back pain, and WHO’s 2023 data emphasized that women aged 80 and above are especially affected by CLBP-related functional limitations.^[5,6]^

Beyond physical discomfort, CLBP also affects sleep and cognitive function.^[7]^ Sleep disturbance (SD) is particularly common among individuals with chronic pain and has been identified as a modifiable mediator between chronic pain and cognitive symptoms such as subjective memory impairment (SMI).^[8–10]^ This triad (chronic pain, SD, and cognitive decline) presents a complex challenge that necessitates integrated intervention strategies.

Aquatic exercise, such as aquarobics, has emerged as a promising non-pharmacological intervention. It offers low-impact physical activity suited to older adults and provides psychological benefits including mood enhancement and stress reduction.^[11,12]^ Aquarobics combines rhythmic water-based movements with music, supporting both physical and cognitive health.^[13,14]^ However, few studies have simultaneously examined its effects on pain-related disability, SD, and SMI in older women with CLBP.

Given its potential to address multiple age-related health issues simultaneously, this study holds clinical significance for researchers investigating holistic interventions, for clinicians seeking safe and accessible treatments for older adults, and for patients aiming to improve quality of life through integrated care.

Therefore, this study aimed to evaluate the effects of a 12-week aquarobic intervention on back pain-related disability, SD, and SMI in community-dwelling older women with CLBP. We hypothesized that the intervention group would show significant improvements in all 3 domains (mobility, sleep quality, and memory) compared to a sedentary control group. Furthermore, we expected that reductions in back pain disability would have both direct and indirect effects on memory, mediated by SD.

## 2. Methods

### 2.1. Study design

This study employed a quasi-experimental, nonequivalent control group pretest-posttest design to evaluate the effects of aquarobic exercise on back pain disability, SD, and SMI in older women. This study was reviewed by C University’s Bioethics Review Committee prior to data collection. Exemption approval (No. 2-1046881-A-N-01-201707–HR-031-09-03) was granted. Due to practical constraints in the community setting, participants were assigned to either the intervention or control group based on their availability and preference. To control for potential baseline differences between groups, analysis of covariance (ANCOVA) was conducted.

The intervention group participated in a structured aquarobic exercise program for 8 weeks, 3 times per week, with each session lasting 60 minutes. Each session consisted of a 10-minute warm-up, 40 minutes of aquarobic training focused on flexibility, balance, and muscle strength, and a 10-minute cool-down. Exercises were led by certified instructors with over 5 years of experience in elderly fitness training. The control group did not participate in any structured exercise program and continued with their normal daily activities.

### 2.2. Study participants

Participants were women aged between 65 and 80 years who had been clinically diagnosed with CLBP lasting for more than 3 months. Eligible participants had not participated in any structured exercise programs within the previous 6 months and were cognitively capable of understanding the study purpose and providing informed consent.

Participants were excluded if they had severe cardiovascular, neurological, or musculoskeletal disorders; diagnosed dementia or significant cognitive impairment; were currently participating in other clinical trials; or were taking medications that could affect pain perception or sleep quality. These factors were excluded to minimize confounding variables and ensure consistency in outcome measurement. Participants were recruited from 2 local community centers in City D, South Korea.

An a priori power analysis was conducted using G*Power 3.1.9.7 (Heinrich-Heine-Universität Düsseldorf, Department of Experimental Psychology, Düsseldorf, North Rhine-Westphalia, Germany) to determine the required sample size for an ANCOVA, with 2 independent groups and one covariate (pretest score). Assuming a medium effect size (*f* = 0.25), an alpha level of 0.05, and a desired power of 0.80, the required sample size was calculated to be 128 participants. To ensure sufficient statistical power and account for potential attrition, a total of 168 participants were recruited (aquarobic exercise group: experimental group, n = 79; sedentary group: control group, n = 89). The control group participated in sedentary programs such as singing, musical instruments, calligraphy, Korean language, and art classes at welfare or community centers. However, 16 female participants (11 from the experimental group and 5 from the control group) were excluded from the final analysis because they either began taking medication or were unable to attend the program consistently during the intervention period. As a result, 152 participants (experimental group, n = 68; control group, n = 84) were included in the final analysis. This sample yielded an estimated statistical power of approximately 0.989. The distribution of study participants is summarized in Table 1.

**Table 1:** Distribution by sociodemographic characteristics.

Variables	Group		Total (n = 152)
	Aquarobics		
	Experimental group (n = 68)	Control group(n = 84)	
Age			
Mean ± SD	70.14 ± 1.48	71.07 ± 1.04	
>65 years old	15	9	24
>70 years old	36	48	84
>75 years old	17	27	44
Education			
Elementary school	9	26	35
Middle school	34	39	73
High school	15	11	26
College	10	8	18
Income level (million won/month)			
Mean ± SD	199.27 ± 22.88	198.46 ± 19.79	
>150	19	28	47
>200	31	39	70
>250	18	17	35
Back pain period (year)			
Mean ± SD	8.68 ± 0.31	8.71 ± 0.48	
>1	9	11	20
>3	27	32	59
>5	32	41	73

### 2.3. Measurement tools

To assess the suitability and usability of the survey content, validity was ensured through an expert meeting involving 1 doctorate in Leisure and Recreation Studies, 1 in Elderly Welfare Studies, and 1 practitioner responsible for managing a senior college. The questionnaire contained 6 questions regarding sociodemographic factors. The Back Pain Disability Index (BPDI) used the Korean version of the Oswestry BPDI, revised to account for Korean cultural characteristics, and validated for the Korean population.^[14]^ It comprises 10 items, excluding 1 question about sexual life, resulting in 9 items scored on a Likert scale ranging from 0 (no disability) to 5 (severe disability). The highest possible score was 45, and the functional disability score was calculated as a percentage. Higher scores indicated more severe disability.

SD was measured using the Korean Sleep Scale A, developed by Oh et al.^[15]^ This tool consists of 15 questions across 4 subfactors: sleep pattern, evaluation, outcome, and disturbance, rated on a 4-point Likert scale. Positive questions were scored inversely, with higher scores indicating greater SD.

SMI was assessed using the Korean version of the Memory Complaint Scale developed by Vale et al.^[16]^ The KMCS includes 7 questions, with scores for each question ranging from 0 to 2. Higher scores indicate a greater degree of memory disorder.

### 2.4. Data collection

Outcome data were collected by trained research assistants who were not involved in the design or delivery of the intervention, ensuring professional objectivity. All assessors were blinded to group allocation to minimize bias. To maintain intervention fidelity, an independent reviewer monitored adherence to the program protocol but was not involved in outcome measurement or data analysis. Data collection occurred at 2 time points: baseline (prior to the intervention) and post-intervention (immediately after the 12-week program). The study was conducted between March 2021 and January 2023 in elderly welfare facilities located in City D. Surveys measuring BPDI, SD, and SMI were administered at the exercise venues (swimming pool and gymnasium) by the principal investigator and a trained assistant. Participants who experienced difficulty with self-reporting due to visual or literacy issues (n = 6) were assisted by research staff, who read questions aloud and recorded responses as indicated. Informed consent forms were attached to each questionnaire to confirm voluntary participation.

Compliance was defined as attending at least 80% of the scheduled sessions. In the intervention group, 90% of participants met this criterion. The dropout rate was 9.5% (n = 16), with most withdrawals attributed to personal or health-related reasons unrelated to the intervention. No adverse events were reported during the study period.

### 2.5. Data analysis

The data collected in this study were analyzed using Statistical Package for the Social Sciences (SPSS) version 26.0 (IBM Corp., Armonk) and Analysis of Moment Structures (Amos) 26.0 (IBM Corp.).

SPSS version 26.0 was used to analyze differences in low BPDI, SD, and SMI between the experimental and control groups. Baseline homogeneity between the experimental and control groups was verified through independent *t*-tests and chi-square tests for demographic and clinical variables. Given the nature of the field research, where pre-measurements between the experimental and control groups differed significantly, a nonhomogeneous control group design was applied. A covariate analysis (ANCOVA) was conducted with pre-measurement sets as covariates to control for these initial differences and examine the main effects. Additionally, multicollinearity was assessed through correlation analysis.

Amos 26.0 was used to identify the causal relationships among the variables using structural equation modeling with covariates. To verify the validity and reliability of the measurement tools used, the relationships between the items were examined using structural modeling theory. Confirmatory factor analysis was conducted to evaluate the appropriateness of the structural model. The absolute fit indices used as model fit indicators were chi-square (*χ*^2^), root mean square residual, goodness-of-fit-index, comparative fit index (CFI), and root mean square error of approximation (RMSEA). The criteria met for all 3 constructs being assessed is provided in Table 2.

**Table 2:** Confirmatory factor analysis results of measurement variables.

Factor	Questionnaire	Standard load	Standard error	C.R.	AVE
Back Pain Disability Index	1	.726	.021	.939	.838
	2	.801	.041		
	3	.761	.030		
	4	.804	.010		
	5	.724	.028		
	6	.703	.039		
	7	.608	.011		
	8	.667	.020		
	9	.734	.013		
*X*^2^ = 41.371, df = 19, GFI = 0.912, TLI = 0.925, CFI = 0.913, RMSEA = 0.069					
Sleep disturbance					
Sleep pattern	1	.801	.021	.859	.803
	2	.811	.054		
	3	.797	.031		
	4	.705	.051		
	5	.719	.070		
	6	.783	.107		
Sleep assessment	1	.807	.213	.892	.843
	2	.799	.136		
	3	.831	.105		
	4	.697	.091		
	5	.728	.020		
Sleep results	1	.802	.091	.901	.891
	2	.786	.075		
Sleep-inhibiting factors	1	691	.165	.867	.811
	2	.795	.076		
*X*^2^ = 50.639, df = 23, GFI = 0.931, TLI = 0.921, CFI = 0.914, RMSEA = 0.080					
Subjective memory impairment	1	.694	.130	.910	.864
	2	.793	.015		
	3	.809	.108		
	4	.776	.064		
	5	.789	.103		
	6	.806	.067		
	7	.826	.009		
*X*^2^ = 48.976, df = 26, GFI = 0.932, TLI = 0.929, CFI = 0.926, RMSEA = 0.083					

CFI = comparative fit index, GFI = goodness-of-fit index, RMSEA = root mean square error of approximation, TLI = Tucker–Lewis index.

The experimental and control groups were compared before and after the experiment with a significance level of *P* < .05.

## 3. Results

### 3.1. Effect of participation in aquarobics on BPDI, SD, and SMI

**Figure 1.**
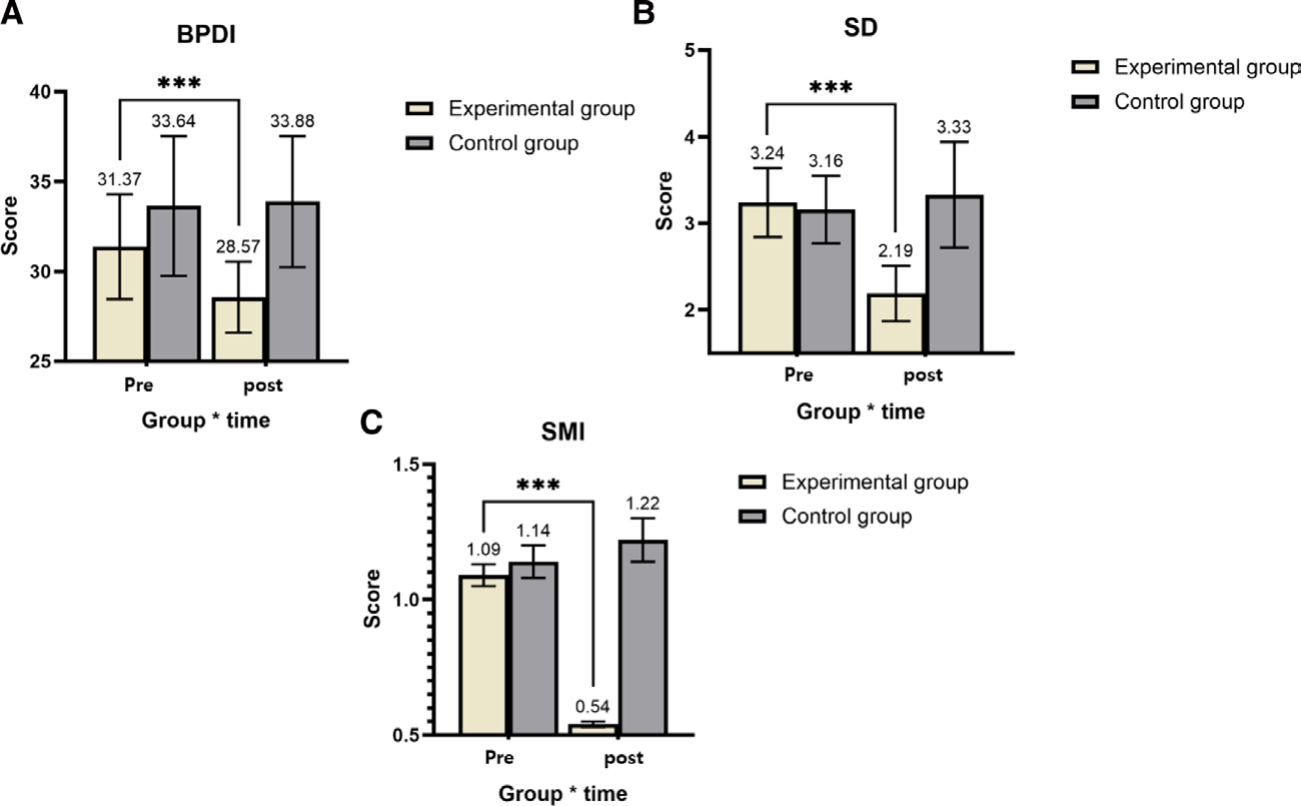
Pretest and post-test of BPDI, SD, and SMI of the groups. (A) Back Pain Disability Index (BPDI). (B) Sleep disturbance (SD). (C) Subjective memory impairment (SMI). ***P < .001.

As presented in Table 3 and Figure 1A, participants in the aquarobic group demonstrated a significant improvement in pain-related disability, with BPDI scores decreasing from 31.37 (SD = 2.91) to 28.57 (SD = 1.97), yielding a mean change of –2.80. In contrast, the control group showed minimal change, with scores slightly changing from 33.64 (SD = 3.89) to 33.88 (SD = 3.64), a mean difference of –0.24. This group difference was statistically significant, *F*(1,149) = 331.517, *P* < .001.

**Table 3:** Means and standard deviations of pretest and posttest of Back Pain Disability Index (BPDI), sleep disturbance (SD), and subjective memory impairment (SMI).

Factor	Group	Pretest	Posttest	*F*
		*M* ± SD	*M* ± SD	
Back Pain Disability Index (BPDI)	Aquarobic class (Experimental group)	31.37 ± 2.91	28.57 ± 1.97	*F* = 331.517***
	Sedentary class (Control group)	33.64 ± 3.89	33.88 ± 3.64	
Sleep disturbance (SD)	Aquarobic class (Experimental group)	3.24 ± 0.40	2.19 ± 0.32	*F = *21.310***
	Sedentary class (Control group)	3.16 ± 0.39	3.33 ± 0.61	
Subjective memory impairment (SMI)	Aquarobic class (Experimental group)	1.09 ± 0.04	0.54 ± 0.01	*F* = 28.617***
	Sedentary class (Control group)	1.14 ± 0.06	1.22 ± 0.08	

*** *P* < .001.

Similarly, SD scores showed a marked improvement in the experimental group, as illustrated in Figure 1B. Scores declined from 3.24 (SD = 0.40) to 2.19 (SD = 0.32), while the control group experienced a slight increase from 3.16 (SD = 0.39) to 3.33 (SD = 0.61). ANCOVA revealed a significant main effect of group, *F*(1,149) = 21.310, *P* < .001 (Fig. 1B).

For SMI, shown in Figure 1C, participants in the aquarobic group improved from 1.09 (SD = 0.04) to 0.54 (SD = 0.01), with a mean change of –0.55. Meanwhile, the control group showed a slight increase from 1.14 (SD = 0.06) to 1.22 (SD = 0.08), a mean change of 0.08. This difference was also statistically significant, *F*(1,149) = 28.617, *P* < .001 (Fig. 1C).

Overall, the results indicate that participation in the aquarobic exercise program significantly improved back pain-related disability, SD, and SMI among older adults compared to the control condition.

### 3.2. Correlation between factors

Pearson product-moment correlation analysis was conducted to examine the relationships between BPDI, sleep-related subfactors, and SMI, as presented in Table 4.

**Table 4:** Results of analysis on correlations between variables.

Factor	1	2	3	4	5	6
	BPDI	SD				SMI
BPDI (1)	1					
Sleep pattern (2)	.437*	1				
Sleep assessment (3)	.449*	.309**	1			
Sleep results (4)	.315**	.286**	.294**	1		
Sleep-inhibiting factors (5)	.438*	.364**	.299**	.104***	1	
SMI (6)	.308**	.312**	.349**	.433*	.366**	1

BPDI = Back Pain Disability Index, SD = sleep disturbance, SMI = subjective memory impairment.

* *P* < .05

** *P* < .01.

*** *P* < .001.

BPDI showed statistically significant positive correlations with sleep pattern (*r* = .437, *P* < .05), sleep assessment (*r* = .449, *P* < .05), sleep results (*r* = .315, *P* < .01), and sleep-inhibiting factors (*r* = .438, *P* < .01). Additionally, BPDI was moderately associated with SMI (*r* = .308, *P* < .05). SMI was also significantly correlated with all 4 sleep subfactors: sleep pattern (*r* = .312, *P* < .01), sleep assessment (*r* = .349, *P* < .01), sleep results (*r* = .433, *P* < .05), and sleep-inhibiting factors (*r* = .366, *P* < .01).

Pearson correlation coefficients across all variables were below 0.60, confirming that there were no concerns of multicollinearity in the dataset.

### 3.3. Causal relationships between variables

The structural relationship analysis examined the relationship between BPDI, sleep disorders, and SMI in older women participating in aquarobics care with the following results.

#### 3.3.1. Verification of model fit

As shown in Table 5, the structural equation model demonstrated an acceptable fit to the data. The model fit indices were as follows: *χ*^2^ = 41.089, degrees of freedom = 22, *χ*^2^/df = 1.868, *P* < .001, Tucker–Lewis index = 0.941, CFI = 0.938, and RMSEA = 0.076. According to conventional cutoff values (*χ*^2^/df < 3.0; Tucker–Lewis index and CFI ≥ 0.90; RMSEA between 0.05 and 0.10), all indices fell within acceptable ranges. These results indicate that the proposed research model fits the observed data reasonably well.

**Table 5:** Index of goodness fit test for the research model.

Overall index	*χ* ^2^	*χ*^2^/df (22)	Sig	TLI	CFI	RMSEA
Research model	41.089	1.868	0.000	0.941	0.938	0.076
Fit index standard		<3.0	>0.05	≥0.9	≥0.9	≤0.05–0.1

CFI = comparative fit index, TLI = Tucker–Lewis index.

#### 3.3.2. Analysis of interpersonal relationships

As shown in Table 6, the direct effects within the structural model included the impact of BPDI on SD (*β* = 0.784), BPDI on SMI (*β* = 0.591), and SD on SMI (*β* = 0.396).

**Table 6:** Structural efficiency.

Path	Direct effect	Indirect effect	Effect
BPDI → SD	0.784	–	0.784
BPDI → SMI	0.591	–	0.591
SD → SMI	0.396	–	0.391
BPDI → SD → SMI	–	.784 × .396	0.310
Total effect	1.771	.310	2.081

BPDI = Back Pain Disability Index, SD = sleep disturbance, SMI = subjective memory impairment.

An indirect effect was also identified, wherein BPDI influenced SMI through the mediating role of SD, with a calculated value of *β* = 0.310 (0.784 × 0.396). The total effect of BPDI on SMI, combining both direct and indirect effects, was *β* = 0.901. The overall cumulative effect across the structural model was *β* = 2.081, indicating a strong combined impact of the predictor variables.

**Figure 2.**
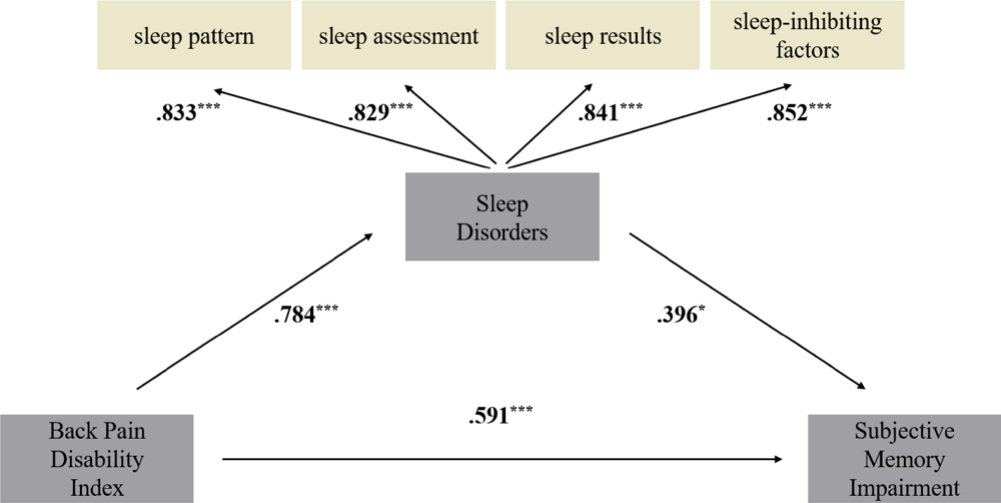
Verification of the structural relationship between Back Pain Disability Index (BPDI), sleep disturbance (SD), and subjective memory impairment (SMI) following participation in aquarobics. It was verified that the BPDI, due to aquarobics participation directly impacted SD and SMI and indirectly impacted SMI through sleep disorders. This demonstrates a causal relationship between BPDI and participation in aquarobics conditions, SD, and SMI. ***P < .001.

These findings confirm that BPDI, influenced by participation in aquarobics, directly affected both SD and memory impairment, while also indirectly influencing SMI through its effect on sleep. This supports the hypothesized causal relationship among BPDI, SD, and SMI. The results of the path analysis are graphically represented in Figure 2.

## 4. Discussion

This study focused on participation in an aquarobics program as an appropriate physical activity for older adult women with chronic back pain and analyzed the relationship between the BPDI, SD, and SMI. Participation in aquarobics significantly reduced BPDI, SD, and SMI.

First, the finding that participation in aquarobics reduces the BPDI aligns with previous studies. Recent studies suggest that managing low back pain through exercise reduces healthcare costs and improves quality-adjusted life years compared to usual care for acute and chronic patients. Khanjari and Kalkhoran^[17]^ investigated the effectiveness of the combination of 12 aquatic exercise sessions and pain neurophysiology education in 55 patients with CLBP. These findings reveal a significant interaction between treatment conditions and pain intensity.^[18]^

Yalfani et al^[19]^ evaluated the effects of a 6-week aquatic exercise program on pain, disability, and function of the trunk and pelvic girdle muscles in 24 women with CLBP. A study on aquatic exercise programs for patients with back pain reported that subjective pain improved,^[19]^ while another study suggested significant improvements in pain, disability, and trunk muscles.^[20]^ Previous research has also suggested that 12 weeks of aquatic exercise increases lumbar strength in middle-aged women with CLBP and improves lower back pain and body composition in older men with lower back pain.^[21]^ Another study reported that pain decreased after aquatic exercise, even in patients with arthritis and difficulties in daily life.^[22]^ These previous studies and the present study suggest that aqua aerobics as part of aquatic exercise is an effective rehabilitation method for reducing back pain in older adult women with CLBP.

Second, aquarobics participation was found to reduce SD in older adult women with CLBP. The few studies that have measured sleep outcomes from aquatic exercise suggest that it may produce greater benefits for sleep than land-based exercise^[11,12,23]^ and have reported positive effects on sleep quality in patients with fibromyalgia performing aquarobics.^[24]^

This decline in sleep quality among older adults has prompted research into interventions such as aquarobics to address SDs, with findings suggesting that exercise is a crucial medium for producing positive effects. Furthermore, the effects of aquarobics on sleep quality have been observed, particularly in women. Older women participating in aquarobics experience significant improvements in sleep quality.^[25]^ Such improvements may be attributed to the enjoyable nature of aquarobics and the fatigue it induces, which support better sleep. Ultimately, water-based activities promote blood circulation, reduce muscle tension, and induce relaxation due to the resistance, pressure, and eddy currents of water, which can foster positive thinking.^[26]^ Therefore, underwater exercise is an effective medium for overcoming SDs by facilitating the recovery of mental function.^[27]^

Third, participation in aquarobics is effective in reducing SMI in older adult women with CLBP. Physical activities that can be incorporated into daily life are cost-effective for older adults and can sustainably enhance cognitive function.^[1]^ A review of previous studies on the relationship between aquatic exercise and older adults’ cognition indicated that aquatic psychological exercise programs effectively enhance cognitive function in community-dwelling older adults.^[2]^ Anecdotal evidence suggests that for those with dementia, water-based exercise has significant behavioral and psychological benefits, with reports suggesting reduced wandering and improved social interactions and sleeping patterns.^[28]^ Underwater interval training effectively improved brain nerve factors (IGF-1) and cognitive function in older adults,^[29]^ supporting the findings of this study.

Considering the findings of previous studies and the results of this study, aquarobics can be considered an active coping strategy that enhances cognitive function and reduces SMI in older adults. However, a limitation remains as water exercise and cognition studies are insufficient, and existing water exercise programs primarily focus on the body through practice and repetitive training. Therefore, programs that stimulate cognition with a focus on spontaneity are required.

Finally, the causal relationship among BPDI, SD, and SMI in aquarobics participants was measured since appropriate physical activity in water can reduce the BPDI scores, alleviating SD and ultimately helping overcome SMI. Previous studies on cognitive function in older adults have shown significant associations with health-related characteristics, such as exercise, and physical ailments, such as back pain and sleep.^[9,10,30]^ The current study indicates that participation in aquarobics, an appropriate physical activity for older adults with CLBP, significantly reduces BPDI scores, which in turn lowers SD and positively affects SMI. A causal relationship exists between the BPDI, SD, and SMI.

SDs caused by pain can lead to various complications, particularly cognitive impairment and functional disabilities, threatening the daily lives and health of older adults.^[31]^ Many patients with chronic pain have sleep difficulties and report poor sleep quality.^[32]^ Numerous studies have reported that pain disrupts sleep and that sleep deprivation and SD increase pain and impair cognitive function, indicating a strong correlation between pain and sleep.^[33]^ SD has consistently been reported as a risk factor and major predictor of cognitive impairment.^[8]^ Therefore, health-related characteristics, such as physical activity and sleep, must be identified to prevent cognitive decline in older adults. Baker et al^[34]^ argued that cognitive function should be a key treatment goal for individuals experiencing chronic pain and that individualized approaches that consider pain and sleep quality related to cognitive function should be devised. Despite its importance, cognitive function enhancement has been overlooked in interventions for older adults with low back pain. Therefore, an integrated management program that includes cognitive function screening and management, along with interventions to alleviate pain and improve sleep, is necessary for older adults with low back pain.

Although the minimal clinically important difference is commonly used to assess clinical relevance, this study focused on community-dwelling older adults rather than clinical patients. Therefore, while the observed improvements were statistically significant, the application of minimal clinically important difference should be interpreted with caution and considered as a supplementary reference. Nevertheless, the findings support the incorporation of aquarobic exercise into non-pharmacological care strategies for older adults with CLBP. Improvements in mobility, sleep, and memory suggest that this intervention may serve as a cost-effective, accessible, and holistic option for both clinicians and patients. Future studies should investigate its long-term effects and broader applicability across diverse elderly populations.

## 5. Conclusion

This study demonstrated that a 12-week aquarobic exercise program significantly reduced back pain-related disability, improved sleep quality, and alleviated SMI in older women with CLBP. Structural equation modeling indicated that pain reduction contributed to cognitive improvement both directly and indirectly through reduced SD. These findings suggest that aquarobics may serve as an effective, accessible, and non-pharmacological intervention to enhance both physical and cognitive health in community-dwelling older adults.

## Author contributions

**Conceptualization:** MoonSook Lee.

**Data curation:** MoonSook Lee.

**Funding acquisition:** Jiyoun Kim.

**Project administration:** MoonSook Lee.

**Writing – original draft:** MoonSook Lee.

**Writing – review & editing:** Jiyoun Kim.
